# Role of antigen-43 on biofilm formation and horizontal antibiotic resistance gene transfer in non-O157 Shiga toxin producing *Escherichia coli* strains

**Published:** 2017-04

**Authors:** Roholla Taghadosi, Mohammad Reza Shakibaie, Reza Ghanbarpour, Hossein Hosseini-Nave

**Affiliations:** 1 Department of Microbiology and Virology, Kerman University of Medical Sciences, Kerman, Iran; 2 Department of Microbiology, Faculty of Veterinary Medicine, Shahid Bahonar University, Kerman, Iran

**Keywords:** Shiga toxin producing *E. coli*, Antibiotic resistance, Biofilm, PCR, Conjugation

## Abstract

**Background and Objectives:**

The objectives of this study were to evaluate the antibiotic resistance profiles, biofilm formation, presence of antigen 43 (*Ag*43) gene, and transfer of antibiotic resistance phenotype among non-O157 Shiga toxin producing *Escherichia coli* (STEC).

**Materials and Methods:**

From October 2014 to November 2015 a total of 276 stool samples were collected from healthy calves, goats and 395 patients with the sign of nonbloody diarrhea and screened for presence of *stx* and serotype O157 genes by polymerase chain reaction (PCR) technique. Susceptibility to 14 antibiotics was determined as per CLSI guideline. Presence of *Ag*43 and intimin (*eaeA*) genes were detected by PCR. Biofilm formation was measured by microtiter plate method. Conjugation was carried out by membrane filter technique.

**Results:**

We isolated 74 (93.6%) non-O157 STEC strains from 41 calves, 33 goats and 5 (6.3%) patients’ stools, however, no O157 serotype was detected in our study. Resistance was observed most commonly to tobramycin (66.2%), kanamycin (48.6%), and amikacin (29.7%) and less frequently to ciprofloxacin (4.1%), amoxicillin-clavulanic acid (5.4%), and ceftriaxone (9.5%) in isolates recovered from calves and goats fecal samples, whereas, all human isolates were sensitive to ceftazidime, ciprofloxacin, tobramycin and imipenem, respectively. Furthermore, Ag43 was detected in 60 STEC isolated from animals and 5 human origins (no *eaeA* gene was found in this study). Biofilm formation from Ag43^+^ and Ag43^−^ colonies showed 20 isolates with strong biofilm activities. Cefotaxime resistance phenotype was transferred to *E. coli* ATCC 25922.1 (Nal^r^) by conjugation at a frequency of 1.6×10^−4^.

**Conclusion:**

From the above results we concluded that, human infections with non-O157 STEC were significantly low in Kerman. Ag43 was insignificant with biofilm quantity in most cases.

## INTRODUCTION

A biofilm is a community of any group of micro-organisms stick to each other on living or non-living surfaces within a self-produced matrix of extracellular polymeric substance (EPS) such as polysaccharides, extracellular DNA and proteins (1). Biofilms have been found to be involved in a wide variety of microbial infections and by one estimate 80% of all infections are caused by biofilm forming bacteria and fungi (2).

A number of different factors have been shown to be important for the ability of a given bacterial strain to form a biofilm. First, flagella assisted motility (twitching motility) enables the cells to reach the surfaces (3), second, adherence factors, for example fimbriae *eaeA* and curli, facilitate attachment and the establishment of initial colonization on the target surface and finally the development of exopolysaccharides matrixes help forming the three-dimensional structure of the biofilm (4). Cell to cell communication, occur via quorum sensing molecules like N-acyl-homoserine lactone assist final structure of the biofilm (5).

Recent studies have shown that non-O157 STEC are emerging as important pathogen associated with numerous human infections as well as outbreaks of food-borne illnesses (6). It estimated that O157 is responsible for 35.9% of STEC infections, whereas non-O157 STEC was responsible for 64.1% of STEC infections in the United States (7). Cattles (calves) are considered the primary reservoir of both O157 and non-O157 STEC without suffering from any pathological symptoms. Wide ranges prevalence of non-O157:H7 STEC in feces from dairy (0.4–74%) and beef (2.1–70.1%) cattles have been reported from various countries (8). Many of the STEC serotypes apparently non-pathogenic and appeared to be lacking one or more virulence factors (9).

It has been reported that biofilm formation occurred in 28% O157 STEC and 51% non-O157 STEC strains from different serotypes and sources, when the assays were performed at 28°C for 48 h (10). Similarly, it has been shown that, biofilm formation by various STEC serotypes on a polystyrene surface was highly strain-dependent, whereas the two non-O157 serotypes showed a higher potency of pellicle formation at air-liquid interfaces on a glass surface compared with serotype O157:H7 (11). Biofilm formation has a great variability among STEC strains and cannot be related to a specific pulsotype nor even to serogroup or presence of virulence genes (12).

Antigen 43 (Ag43) is a self-recognizing surface adhesin found in most *E. coli* strains. Due to its excellent cell-to-cell aggregation characteristics, Ag43 confers clumping and fluffing of cells and promotes biofilm formation. Ag43 expression is repressed by the cellular redox sensor OxyR (13). Similarly, Ag43 is also a member of the autotransporter family of excreted proteins within a single peptide chain. It is processed into a mature form consisting of two subunits, α and β (14). Both receptor recognition and receptor target is provided in the same polypeptide and has been found to induce characteristic surface properties on host cells, such as autoaggregation and frizzy colony morphology. It was further established that autoaggregation of cells takes place through an Ag43–Ag43 interaction and follows first order kinetics in a cell density dependent manner (14). A key function of Ag43 seems to be the promotion of bacterial biofilm formation (15). A study by Danese et al. (16) showed that, Ag43 contributed to *E. coli* biofilm formation in glucose minimal medium, but not in Luria-Bertani broth.

Furthermore, flagella, motility and type IV pilus-based twitching motility have been shown to be required for the initial attachment and development of a biofilm by *P. aeruginosa* and *E. coli* (17,18). Obviously, cells inside a biofilm do not require extensive motility until the time they leave to colonize another available surface. Mutations that disrupt twitching mediated motility inhibit the transition of free swimming cells of various bacterial species into biofilms (18). Despite prior implications in biofilm function, the particular aspect of flagellar biogenesis and function that is needed for biofilm formation in any species of bacteria had not yet been clearly defined. It has been suggested that flagella could function in a direct fashion by physically adhering to an abiotic surface (18).

Plasmid mediated horizontal transfer of multi-drug resistance (MDR) traits plays a key role in the dissemination of antimicrobial resistances among STEC isolates both in veterinary and medical practice. Multiple plasmids belonging to major replicon types such as IncF, and IncA/C, are associated with this phenomenon. It has been suggested that overuse of antibiotics as food additive in animal husbandry creates a threat to human and veterinary by induction of antibiotic resistance and subsequent transfer to sensitive enteric normal flora via plasmid mediated conjugation process (19).

Recently we reported the role of quorum sensing factor N-Acyl homoserine lactone in biofilm forming uropathogenic *E. coli* isolated from urinary tract infection samples (5). The present study was performed to determine the role of Ag43, and *eae* genes on biofilm formation and horizontal antibiotic resistance gene transfer in non-O157 Shiga toxin-producing *E. coli* strains isolated from human and animal fecal samples for the first time.

## MATERIALS AND METHODS

### Bacterial isolates.

A total of 79 non-O157 STEC were isolated from the stool samples of 156 calves and 120 goats and 395 human feces. The human isolates collected from the rectal swabs of the patient’s with nonbloody diarrhea referred to two referral hospitals and one private laboratory in Kerman city, southeast region of Iran. Duplicate samples were collected from each patient by an expert laboratory technician. All stools from animal sources collected by veterinarians from School of Veterinary Medicine, Shahid Bahonar University in Kerman and transferred to the microbiology laboratory, Kerman University of Medical Sciences. Samples were initially inoculated on MacConkey and Eosin Methylene blue (EMB) agar plates (Merck, Darmstadt, Germany) and incubated at 37°C for 24 h. The individual colonies were then examined by various biochemical and conventional diagnostic tests (5). The *E. coli* isolates were then screened for presence of *stx* and O157 genes by PCR using specific primers pairs as described before (19).

### Antimicrobial susceptibility testing.

Antibiotic sensitivity of the STEC isolates to 14 antibiotics was determined by Kirby-Bauer disk diffusion breakpoint method on Mueller-Hinton agar (Hi-Media, Mumbai, India) using commercially available paper disks (Mast corporation Ltd. UK). The sensitivity was interpreted for each antibiotic according to Clinical and Laboratory Standards Institute (CLSI-2012) criteria (20). Antimicrobial agents tested were: Aztreonam (ATM; 30), amikacin (AK; 30), piperacillin (PRL; 100), augmentin (AUG; 30), kanamycin (K; 30), ciprofloxacin (CIP; 5), ceftriaxone (CRO; 30) ceftazidime (CAZ; 30), cefotaxime (CXM; 30), tobramycin (TN; 10), nalidixic acid (NA; 30), trimetho-prime-sulfametoxazole (TS; 25), ampicillin (AP; 10) and imipenem (IMI; 10). *E. coli* ATCC 25922 was used as standard sensitive strain.

### Biofilm formation assay under static condition.

Biofilm formation on the abiotic surface was quantified according to the method described by O’Toole et al. (18). Briefly, 1:100 diluted STEC strains were inoculated into a 96 well microtiter plate containing 100 μl fresh Tryptic Soy Broth (TSB) supplemented with 0.2% glucose. Growths were monitored after 24 h incubation at 37°C. Unbound cells were removed by inversion of the microtiter plate. Adhered cells were subsequently stained by the addition of 200 μl of 0.1% crystal violet. The stain was removed by thorough washing with phosphate-buffered saline (PBS) and the wells allowed drying. Quantification of cells in biofilms was carried out by solubilization of crystal violet with 200 μl of ethanol. Optical density (OD) at 480 nm was then determined for each isolate. Simultaneously, *P. aeruginosa* PAO1 was used as biofilm positive strain. Data for biofilm formation of all strains were compared with the data for the negative control by Student’s t-test.

### Detection of *Ag*43 and *eaeA* genes by PCR.

To detect the presence of two biofilm related genes Ag43 and *eaeA*, we performed PCR assay as described previously (15). For this purpose, each STEC positive colony was inoculated on Tryptic Soy Agar (Merck, Germany) and incubated for 24 h at 37°C. Two identical colonies were then selected and suspended separately in 500 μl of sterile distilled water in DNase-free microfuge tubes (Eppendorf, Germany) and were incubated at 95°C for 10 min in a water bath. The cell lysates were then centrifuged at 10.000 rpm for 10 min and supernatants and genomic DNAs were used for detection of antigen 43 and intimin (*eaeA*) gene by PCR technique using following primer pairs sequences (Ampliqon, Denmark); Ag43 forward: 5′-ACGCACAACC ATCAATAAAA-3′, Ag43 reverse: 5′-CCCCGCCT CCGATACTGAATGC -3′; *eaeA* forward: 5′-CTGAACGGCGATTAC GCGAA-3′ and *eaeA* reverse: 5′-CCAGACG AT ACGATCCAG-3′ as described before (21). The size of primers was 620 and 917 bp, respectively. The reaction mixtures were prepared as follow; 12.5 μl master mix (Ampliqon, Denmark), 10.5 μl D/W, 0.5 μl each primer, 1 μl template DNA in a total volume 25 μl. The PCR processes were carried out using a temperature gradient thermal cycler (Biometra-T300, Gottingen, Germany). The amplification program consisted of an initial denaturation at 95°C for 5 min, 95°C 30 cycles for 30 s, annealing temperature 57°C for 30s, extension at 72°C for 45s and final extension at 72°C for 5 min. The amplified product was then electrophoresed by using 1.5% agarose gel (Sina-Clon, Iran) prepared in TBE-buffer [89 mM Tris-borate, 2 mM ethylenediamine tetra acetic acid (EDTA), pH 8.0)] for 1 h, stained with UV illuminating dye (green viewer) and image visualized by UV-gel documentation system (Oxford, UK).

### Horizontal antibiotic resistance gene transfer.

Conjugation was performed by membrane filter technique as described previously (22). Here, cefotaxime resistant STEC 1050 and 1152 strains were selected as donor strains, while plasmid-free nalidixic acid resistant mutant of *E. coli* ATCC 25922.1 (Nal ^r^ ) selected as recipient strain (spontaneous Nal ^r^ colonies were generated by gradient plate technique). Conjugation was carried out by mixing 2 ml of donor and 3 ml of recipient cells in a sterile Petri -plate, the mixture was passed through a conjugation assembly. The membrane filter (Sartorius, Germany) was then removed and placed on Tryptic Soy agar (TSA) plate at 37°C for 24 h. Mating was disrupted by vigorous vortexing of the membrane in 5 ml D/W and serially diluted (10 ^−2^ – 10 ^−8^ ) with 5 ml normal saline. 200 μl of each dilution was spread on TSA medium selective for transconjugants (containing 25 μg of cefotaxime per ml and 100 μg of nalidixic acid per ml) and recipient cells (containing 100 μg of nalidixic acid per ml). Colonies that grew on selective agar plates were considered transconjugants. The frequency of conjugation was then calculated as number transconjugants colonies divided by number of recipient multiply dilution factor. Simultaneously, control for donor and recipient strains were carried out to check the presence of any spontaneously developed mutants.

## RESULTS

### Bacterial sources.

In a prospective study, from October 2014 to November 2015, we isolated a total of 395 *E. coli* stool samples from suspected infectious gastroenteritis patients with nonbloody diarrhea and 276 from healthy calves and goats stools. Screening of the *E. coli* isolates for the presence of *stx* gene by PCR in both animal and human origins revealed a total of 79 positive non-O157 STX producing isolates (74 in animals and 5 in human stools) from collected samples. PCR test for O157 antigen using O157 primer pairs was negative for all the cases.

### Antimicrobial susceptibility.

**Fig. 1.**
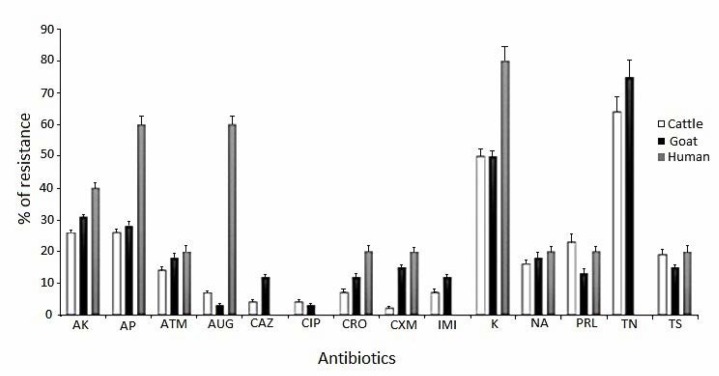
Antibiotic resistant profile of non-O157 STEC isolates in fecal samples of calves, goats and humans origins. Abbreviations: AK= amikacin, AP= ampicillin, ATM= aztreonam, AUG= amoxicillin/clavulanic acid, CAZ= ceftazidime, CIP= ciprofloxacin, CRO= ceftriaxone, CXM= cefotaxime, IMI= imipenem, K= kanamycin, NA= nalidixic acid, PRL= piperacillin, TN= tobramycin, TS= trimethoprim/sulfa methoxazole. Antibiotic resistance was performed at CFU 1 × 10^8^ inoculum size in Muller-Hinton agar.

The results of antimicrobial susceptibility test by disk diffusion breakpoint method are shown in Fig. 1. As the results indicated, 64.5% of the non-O157 STEC strains were resistant to tobramycin, 51.8% to kanamycin, 17.7% to trimethoprim-sulfamethoxazole and 29% to ampicillin, whereas, ceftazidime and ciprofloxacin were the most effective antibiotics evaluated in this study. Similar results were obtained for STEC isolates collected from goat’s fecal samples (Fig. 1). All STEC human isolates were sensitive to ceftazidime, ciprofloxacin and imipenem, but were resistant to kanamycin, ampicillin and amoxicillin/clavulanic acid, respectively (Fig. 1).

### Evaluation of Ag43, *eaeA* and biofilm formation.

**Fig. 2.**
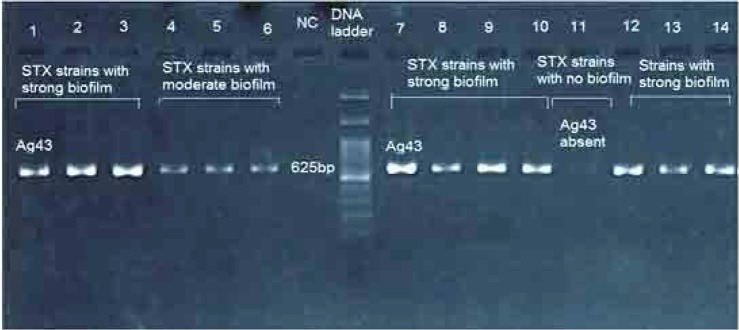
Agarose gel electrophoresis of PCR product of amplified antigen 43 autoadhesin gene detected in non-O157 STEC isolates from animal and human origins. Lanes 1 to 14 indicates strain numbers. DNA marker was consisting of a ladder with 100bp. Electrophoresis was conducted in 1.5 % agarose gel.

**Fig. 3.**
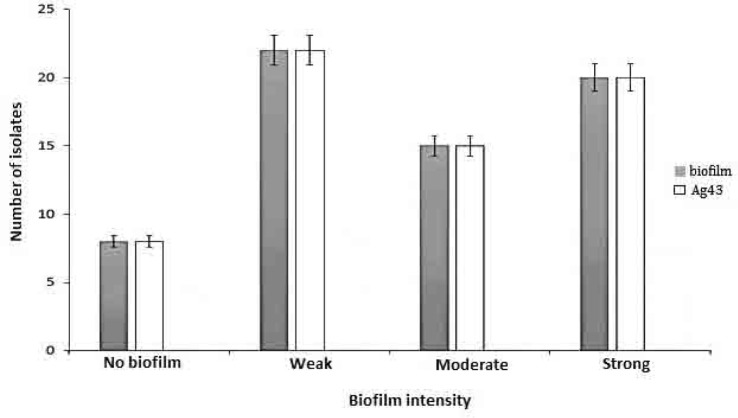
Biofilm quantification, and presence of Ag43 in non-O157 STEC strains isolated from stools of healthy calves, goats and patients with mild diarrhea. *P. aeruginosa* PAO1 was used as positive control.

Agarose gel electrophoresis of PCR amplification of Ag43 gene among STX producing *E. coli* is presented in Fig. 2. As the results indicated, Ag43 gene was detected in 60 animal and 5 human isolates. No intimin gene was detected in the present study. We further examined the biofilm formation quantitatively in those isolates encoded Ag43 (PCR-positive samples) and in those that Ag43 was absent (PCR-negative samples). The results showed that, 8 STEC isolates demonstrated no biofilm, 22 showed weak biofilm, 15 exhibited moderate biofilm and 20 strains showed strong biofilm activity (Fig. 3). There was no any meaningful relationship between biofilm quantity and presence of Ag43.

### Gene transfer experiments.

An attempt was made to transfer cefotaxime resistance gene by conjugation. We performed a filter mating experiment using two STEC 1050 and 1152 as donors and *E. coli* ATCC 25922.1 [Nal ^r^ ] strain as a recipient. The former *E. coli* was resistance to the third generation of cephalosporins. Transconjugants were selected on TSA medium containing 25 μg/ml cefotaxime and 100 μg/ml nalidixic acid as shown in Table 1. Cefotaxime resistance has transferred a frequency of 1.6 × 10 ^−4^ . However, conjugation transfer of cefotaxime resistance phenotype from STEC 1152 strain to *E. coli* ATCC 25922.1 Nal ^r^ was below limits of detection. The transconjugants colonies exhibited cross-resistance to ceftazidime and cefoxitin simultaneously (absence of zone inhibition surrounding antibiotic disks).

**Table 1.** Horizontal transfer of antibiotic resistance between cefotaxime resistance strains of non-O157 STEC as donor and *E. coli* ATCC25922.1 (Nal^r^) as recipient.

**Donor**	**Recipient**	**Selective medium**	**Frequency of conjugation**
STEC 1050	*E. coli* ATCC 25922.1 Nal^r^	Nal (100 μg/ml) + CXM (25 μg/ml)	1.6 ×10^−4^
STEC1152	*E. coli* ATCC 25922.1 Nal^r^	Nal (100 μg/ml) + CXM (25 μg/ml)	–

Nal= nalidixic acid, CXM= cefotaxime. Conjugation frequency calculated as number transconjugants colonies divided by recipient colonies multiply dilution factor. Simultaneously, controls for both donor and recipient were carried out to check the presence of spontaneously developed mutants.

## DISCUSSION

Several studies have reported that flagella are necessary for biofilm formation by *E. coli* and other bacteria such as *Listeria monocytogenes* and *Yersinia enterocolitica* (17, 25). Furthermore, it has been suggested that twitching motility allows improved access to surfaces in the initial attachment. In low-shear environments, however, motility has been shown to have no effect on attachment of *E. coli* to glass (17). It has long been known that *E. coli* can adhere to surfaces via flagella (25), and somewhat more recently, several pathogenic strains have been shown to adhere to epithelial tissue using flagella mediated adhesion (27, 28). Cell-cell aggregation is a phenotype classically associated with Ag43 expression. In *E. coli* K-12, the expression of Ag43 is phase variable due to the concerted action of the Dam methyltransferase (positive regulation) and OxyR (negative regulation) (28).

In this study, we tried to address the role of Ag43, and intimin on biofilm formation and also to determine the frequency of transfer of antibiotic resistant phenotype among non-O157 STEC strains for the first time in Iran. We selected STEC isolates from calves, goats and human with mild diarrhea. Fortunately, majority of non-O157 strains were sensitive to antibiotics used for animal and human medicine. Ceftazidime, ciprofloxacin and trimethoprim-sulfamethoxazole are still good antibiotics for treatment of cattles, goats and human diarrheal infections. A few strains (1050, 1152) were MDR and therefore we used them for gene transfer study. Cefotaxime resistance property was transferred from the cefotaxime resistance STEC strain 1050 to *E. coli* ATCC 25922.1 (Nal ^r^ ). Cross resistance assessment revealed that, the transconjugants were also resistant to ceftazidime and cefoxitin. Our results were consistent with recent study by Schroeder et al. (19) on 23 STEC isolated from animals (mainly cattle, sheep, pigs and goats) between 2005 and 2007 that showed the highest levels of resistance to ampicillin, streptomycin, sulfonamides and tetracycline (19).

We also studied the presence of Ag43, and intimin, we found that, 60% of animal and all human isolates carried Ag43 gene but intimin was absent in STEC population. Further analysis of biofilm formation in strains encoded Ag43 revealed that 35 isolate carried strong and moderate biofilm activity. However, presence of Ag43 had no significant effect on biofilm quantity. It has been demonstrated that activity of Ag43 is associated with the early stages of biofilm development (29). But, Ag43 has also been shown to be dispensable for biofilm formation as it can be replaced by alternative factors, such as conjugative pili (30).

## CONCLUSION

In general, studies concerning non O157-STEC biofilms highlighted that their formation is heterogeneous and mostly dependent on the strains and the conditions used and not dependent to Ag43 at least in complex medium such as Tryptic Soy agar (17). However, there is limited information about the composition of the STEC biofilm matrix. Moreover, non-O157 isolates continue to gain importance as pathogens of concern. Their biofilm forming abilities were shown to be different from those of O157 isolates (10).
